# Development of a Fingertip Glove Equipped with Magnetic Tracking Sensors

**DOI:** 10.3390/s100201119

**Published:** 2010-01-29

**Authors:** Chin-Shyurng Fahn, Herman Sun

**Affiliations:** Department of Computer Science and Information Engineering, National Taiwan University of Science and Technology, Taipei 10607, Taiwan; E-Mail: herman_sun@sitronix.com.tw

**Keywords:** data glove, fingertip tracking, magnetic induction, sensor module, motion constraint, data glove calibration, man-machine interface

## Abstract

In this paper, we present the development of a data glove system based on fingertip tracking techniques. To track the fingertip position and orientation, a sensor module and two generator coils are attached on the fingertip and metacarpal of the corresponding finger. By tracking the fingertip, object manipulation tasks in a virtual environment or teleoperation system can be carried out more precisely, because fingertips are the foremost areas that reach the surface of an object in most of grasping processes. To calculate the bending angles of a finger, we also propose a method of constructing the shape of the finger. Since the coils are installed on the fingertips and metacarpals, there is no contact point between the sensors and finger joints. Hence, the shape of the sensors does not change as the fingers are bending, and both the quality of measurement and the lifetime of the sensors will not decrease in time. For the convenience of using this glove, a simple and efficient calibration process consisting of only one calibration gesture is also provided, so that all required parameters can be determined automatically. So far, the experimental results of the sensors performing linear movement and bending angle measurements are very satisfactory. It reveals that our data glove is available for a man-machine interface.

## 1. Introduction

Interest in studying man-machine interfaces has continued to grow, especially for immersive virtual environment applications and for input devices for portable machines. To achieve more realistic object manipulation, glove-based input devices are commonly chosen as the human-machine interfaces of virtual reality (VR) applications. The data glove is a multi-sensory device that generates a large amount of data and is more complex than other input devices. Nonetheless, most researchers still adopt this device because the natural interfacing characteristic of the data glove with human being is the way to improve system manipulations that are applicable in many specific fields. At present, the data glove has been increasingly employed in the areas of teleoperations and robotic control [1–3], surgery training of medical applications [4,5], entertainment sports (VR systems) [6,7], industrial manufacturing of CAD/CAM applications [8,9], text input devices [10–12], and so on.

The hand-tracking gloves currently marketed include: Sayre Glove, MIT LED Glove, Digital Data-Entry Glove, DataGlove, Dexterous HandMaster, Power Glove, CyberGlove, VPL Glove, and Space Glove [13]. Nowadays, several kinds of sensing technologies have been realized and applied to the development of data gloves. Most of these data gloves provide high accuracy, high reliability, and high capability in measuring the degree of freedom (DOF) of human hands [14–17]. Most of them are constructed using sensors that measure the bending angles of fingers. Although the glove sensors can measure all of the bending angles precisely, the precision of object grasping in the virtual environment is not guaranteed due to the variation in size of a user’s hand wearing the data glove.

The gloves are also mostly built with flex sensors attached on the finger joint positions of the hand. When the fingers are bent, the sensors are also bent and the generated outputs are measured. Based on these outputs, the bending angles of the fingers are calculated. When users wear the data gloves, the stretching and bending of the finger joints occur very frequently. This reduces the lifetime of the sensors and the accuracy of measurements. According to the sensor outputs, the data gloves can be grouped into two classes: one type produces linear outputs, and another produces nonlinear outputs. Either linear or nonlinear data gloves should be calibrated before they are activated in the particular applications. Compared to linear data gloves, the calibration process of nonlinear data gloves is not so easy, owing to the lack of output references of nonlinear sensors [18]. The following depicts the most commonly used sensors for hand tracking applied to the development of glove-based input devices:

- Acoustic tracking sensor
  This kind of sensor uses high frequency audio signals to track the movements of fingers. Such sensors may suffer from acoustic reflections if they are surrounded with hard walls or other acoustically reflective surfaces.
- Optical tracking sensor
  This sensor generally uses an LED or infra-red signal as the source which is conducted toward a transmission media like flexible tubes or fiber optics. Then a photocell sensor is placed at the other end of the media to measure the intensity of the signal.
- Magnetic tracking sensor
  It uses a source element radiating a magnetic field and a small sensor that reports its position and orientation with respect to the source. The magnetic field may be interfered by metallic objects in the environment, which decrease the accuracy of measurement.
- Resistance tracking sensor
  This sensor uses a variable resistance material whose resistivity is varied according to the bending degree of the sensor.

In order to achieve high capability in measuring the DOF of a hand, a lot of sensors should be attached to the data glove, each of which is usually installed on the finger joint position. Low cost data gloves that contain few sensors are available today on the market. However, this kind of data gloves can only measure a few DOF of the hand, so it does not appropriately act as an input device for most of the VR systems that require a high DOF of the hand.

In this paper, we present the development of a sensory data glove that contains only five sensor modules and one sensor coil, but still possesses the ability to measure high numbers of DOF of the hand. Instead of measuring the bending angles of finger joints directly, we track the positions and orientations of fingertips with respect to the metacarpals, so that there is no direct contact point between sensors and finger joints, and thus, the quality of measurement and the lifetime of the sensors will not decrease in time. We also propose a method for constructing the shape of the finger, and based on this shape, the bending angle of the finger joints can be calculated. By measuring the positions of the fingertips, the object grasping process in a virtual environment can be performed more precisely, because in most of such processes, the fingertips are the foremost areas that reach the surface of an object. Using magnetic induction technology, we constructed the data glove sensors, which provide high accuracy of measurement. To avoid the interference of metallic objects in the environment, the sensors and the electromagnetic sources on the glove are arranged in such a way to ensure that the distances between them are near enough, so that the measurement error caused by metallic objects can be ignored.

In the light of the magnetic induction theory, the position and orientation of a single coil sensor can be uniquely described by three position and two orientation parameters. To solve these parameters, a minimum of five equations are needed. If more equations are incorporated, more sensors should be added such that the complexity of the data glove system will increase. To reduce the number of sensors, we design a sensor module that can be used for measuring the fingertip position. The positions of the sensors on the data glove are carefully arranged and the motion constraints among the fingers and the finger joints of the hand [19] are also investigated. The theoretical formulation of the fingertip positions, abduction angles, and the calibration equations are derived directly from the positions and orientations of the sensors in which the motion constrains of the hand are applied.

To make the glove easy to use, a simple and efficient calibration process consisting of only one calibration gesture is proposed, too. The parameters required for calculating the fingertip positions are immediately determined from the calibration equations that are also derived from the positions and orientations of the sensors on the data glove. In the realization of the data glove, ten generator coils arranged into five groups are attached on the metacarpals, while five associated sensor modules are installed on the fingertips of the hand. Additionally one sensor coil is placed on the metacarpal of the middle index for measuring the abduction angles. These ten generator coils tend to interfere with each other if they are not appropriately controlled, and all of the sensors will not produce accurate results. To overcome this problem, three scanning methods are evaluated and the time division technique is eventually adopted in developing the prototype of our data glove.

## 2. The Construction of the Data Glove Sensor

**Figure 1.**
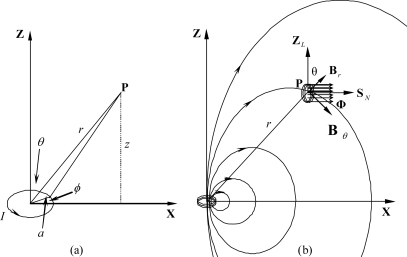
Illustration of magnetic fields: (a) a point **P** located at distance r from a magnetic dipole; (b) the magnetic flux density linked by the coil at position **P** (adapted from [20]).

The sensor of the data glove we have developed is made of small coils (the sensor coils) that are installed on the fingertip positions of the hand. The magnetic flux generators are also made of small coils (the generator coils) activated by sinusoidal waveform generators. Each generator coil may be represented as a magnetic dipole that can be modeled by a single analytical equation. The magnetic dipole is a current loop whose dimensions are much smaller than the distance between the loop and an observation point. The magnetic field at point **P** with distance *r* from a circular dipole as shown in Figure 1a is given by:

(1) $$\begin{array}{l} B_{r}=\frac{\mu_{0}\;{\mathrm{NIa}}^{2}}{4}\;\frac{2\;\text{cos}\;\theta }{r^{3}}\\ B_{\theta }=\frac{\mu_{0}\;{\mathrm{NIa}}^{2}}{4}\;\frac{\text{sin}\;\theta }{r^{3}}\\ B_{\phi }=0 \end{array}$$

where *B_γ_*, *B_θ_* and *B_ϕ_* are the magnetic fields in the directions *a_γ_*, *a_θ_*, and *a_ϕ_* of the spherical coordinates system, respectively, *μ_0_* = 4*π* × 10^−9^ H/cm is the magnetic permeability of a free space, *N* is the number of turns, *I* is the coil current, *a* is the radius of the coil, and *r* >> *a.*

**Figure 2.**
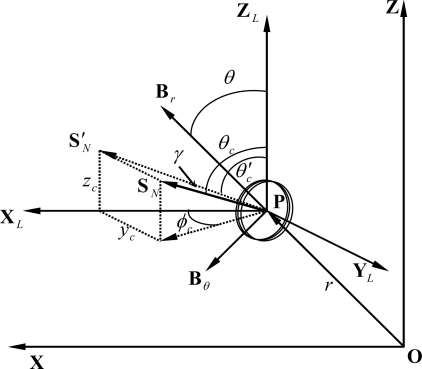
The coil at position **P** with the orientation pointed by unit vector S_N_ (adapted from [20]).

When a single coil sensor is placed at point **P** where the magnetic field intensity is varied in time, the electromotive force (*Emf*) induced in the coil is expressed by the following equation:

(2) $$\mathrm{Emf}=-N_{c}\;\frac{d\Phi }{\mathrm{dt}}$$

where *ϕ* is the magnetic flux density linking the coil and *N_c_* is the number of turns of the coil. The negative sign indicates the direction of the induced current opposite to that of the change of the magnetic flux density. The magnetic flux density linked by the coil depends on the position and orientation of the coil in the magnetic field, as Figure 1(b) shows. Since *B_ϕ_ = 0*, we can assume that the position of point **P** is always in the *xz*-plane. The orientation of the coil at point **P** can be represented by *θ_c_* measured from the *Z_L_*-axis and *ϕ_c_* measured from the *X_L_*-axis as shown in Figure 2, where *X_L_*, *Y_L_*, and *Z_L_* represent the local coordinates system of the coil.

The magnetic field intensity in the direction *S_N_* can be calculated by projecting *S_N_* into the *xz*-plane. The procedure of calculating this magnetic field intensity is stated below:

(3) $$\begin{array}{c} S_{N}^{\prime }=\sqrt{1-{\text{sin}}^{2}\;\theta_{c}\;{\text{sin}}^{2}\;\phi_{c}}=\text{cos}\;\gamma \\ \text{cos}\;\theta_{c}^{\prime }=\frac{\text{cos}\;\theta_{c}}{S_{N}^{\prime }}=\frac{\text{cos}\;\theta_{c}}{\text{cos}\;\gamma }\\ \text{sin}\;\theta_{c}^{\prime }=\frac{\text{sin}\;\theta_{c}\;\text{cos}\;\phi_{c}}{S_{N}^{\prime }}=\frac{\text{sin}\;\theta_{c}\;\text{cos}\;\phi_{c}}{\text{cos}\;\gamma }\\ B_{S_{N}^{\prime }}=B_{r}\;\text{cos}\left(\theta_{c}^{\prime }-\theta \right)+B_{\theta }\;\text{cos}\left(90{}^{\circ}-(\theta_{c}^{\prime }-\theta )\right)\\ =\frac{\text{cos}\;\theta_{c}\;\left(B_{r}\;\text{cos}\;\theta -B_{\theta }\;\text{sin}\;\theta \right)+\text{sin}\;\theta_{c}\;\text{cos}\;\phi_{c}\;\left(B_{r}\;\text{sin}\;\theta +B_{\theta }\;\text{cos}\;\theta \right)}{\text{cos}\;\gamma }\\ B_{S_{N}}=B_{S_{N}^{\prime }}\;\text{cos}\;\gamma =\text{cos}\;\theta_{c}\;\left(B_{r}\;\text{cos}\;\theta -B_{\theta }\;\text{sin}\;\theta \right)+\text{sin}\;\theta_{c}\;\text{cos}\;\phi_{c}\;\left(B_{r}\;\text{sin}\;\theta +B_{\theta }\;\text{cos}\;\theta \right) \end{array}$$

Then the magnetic flux density linked by the coil with radius *b* yields:

(4) $$\Phi ={\pi b}^{2}\left(\text{cos}\;\theta_{c}\;\left(B_{r}\;\text{cos}\;\theta -B_{\theta }\;\text{sin}\;\theta \right)+\text{sin}\;\theta_{c}\;\text{cos}\;\phi_{c}\;\left(B_{r}\;\text{sin}\;\theta +B_{\theta }\;\text{cos}\;\theta \right)\right)$$

By substituting B_γ_ and B_θ_ from (1) into (4), the magnetic flux density becomes:

(5) $$\Phi =\frac{{\pi \mu }_{0}{\mathrm{Na}}^{2}b^{2}I}{{8r}^{3}}\;\left(\text{cos}\;\theta_{c}\;\left(3\;\text{cos}\;2\theta +1\right)+3\;\text{sin}\;\theta_{c}\;\text{cos}\;\phi_{c}\;\text{sin}\;2\theta \right)$$

From (2), the induced electromotive force can be written as the function of the position in the *yz*-plane and the orientation of the sensor coil. This equation yields:

(6) $$\mathrm{Emf}(r,\theta ,\theta_{c},\phi_{c})=-\frac{k}{r^{3}}\;\frac{\mathrm{dI}}{\mathrm{dt}}\left(\text{cos}\;\theta_{c}\;(3\;\text{cos}\;2\theta +1)+3\;\text{sin}\;\theta_{c}\;\text{cos}\;\phi_{c}\;\text{sin}\;2\theta \right)$$

where $k=\frac{{\pi \mu }_{0}N_{c}{\mathrm{Na}}^{2}b^{2}}{8}$.

**Figure 3.**
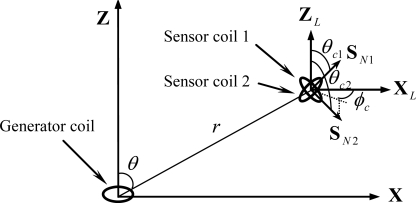
Illustration of the sensor module.

In the development of the data glove, two identical sensor coils are combined to produce a sensor module. These two coils are arranged such that they are co-centered and perpendicular, as illustrated in Figure 3.

Since the two sensor coils are perpendicular, the orientation of the second sensor coil can be expressed as:

(7) $$\theta_{c2}=\theta_{c1}+90{}^{\circ}$$

By substituting (7) into (6), the square of the *Emf* signals produced by the two sensor coils become:

(8) $$\begin{array}{c} {\mathrm{Emf}}_{1}^{2}=\frac{1}{r^{6}}{\left(k\frac{\mathrm{dI}}{\mathrm{dt}}\right)}^{2}(W^{2}\;{\text{cos}}^{2}\;\theta_{c1}+3W\;\text{sin}\;2\theta_{c1}\;\text{cos}\;\phi_{c}\;\text{sin}\;2\theta +9\;{\text{sin}}^{2}\;\theta_{c1}\;{\text{cos}}^{2}\;\phi_{c}\;{\text{sin}}^{2}\;2\theta )\\ {\mathrm{Emf}}_{2}^{2}=\frac{1}{r^{6}}{\left(k\frac{\mathrm{dI}}{\mathrm{dt}}\right)}^{2}(W^{2}\;{\text{sin}}^{2}\;\theta_{c2}-3W\;\text{sin}\;2\theta_{c2}\;\text{cos}\;\phi_{c}\;\text{sin}\;2\theta +9\;{\text{cos}}^{2}\;\theta_{c2}\;{\text{cos}}^{2}\;\phi_{c}\;{\text{sin}}^{2}\;2\theta ) \end{array}$$

where *W* = 3cos2*θ* + 1. Consequently, the power *Emf* signal of the sensor module, which is defined as the sum of the square of the *Emf* signals of the two sensor coils with respect to the generator coil, can be derived from (8) and listed in the following:

(9) $$\mathrm{EmfM}={\mathrm{Emf}}_{1}^{2}+{\mathrm{Emf}}_{2}^{2}=\frac{1}{r^{6}}{\left(k\frac{\mathrm{dI}}{\mathrm{dt}}\right)}^{2}\left({\left(3\;\text{cos}\;2\theta +1\right)}^{2}+9\;{\text{cos}}^{2}\;\phi_{c}\;{\text{sin}}^{2}\;2\theta \right)$$

If *ϕ_c_* = 0, the equation above can be further simplified as:

(10) $$\mathrm{EmfM}=\frac{2}{r^{6}}{\left(k\frac{\mathrm{dI}}{\mathrm{dt}}\right)}^{2}\left(3\;\text{cos}\;2\theta +5\right)$$

## 3. The Construction of the Data Glove

To track the fingertip positions efficiently, the orientations and locations of the sensor modules associated with the generator coils should be carefully arranged on a user’s hand. Instead of placing them on the finger joint positions, the sensor modules are attached on the fingertips and the generator coils are placed near to the metacarpophalangeal (MCP) joints on the dorsal surface of the metacarpals. The distances between the generator coils and sensor modules are close enough such that the potential interference caused by a metallic object with the distance greater than 20 cm from the data glove can be ignored.

Owing to the highly articulated characteristic of a human hand, it possesses approximately 30 DOF that produce almost all hand gestures. Although the hand has so many DOF, the movements of the fingers, however, are highly constrained, so that it cannot make arbitrary gestures. There are many examples of such constraints, for instance, fingers cannot bend backward too much. The constraints that are applicable to simplifying the development of our data glove typically can be classified as follows:

- Intrafinger constraints
  It is the constraint between two joints of the same finger. By applying these constraints, the index, middle, ring, and pinkie distal interphalangeal (DIP) joint movements can be approximated by the following equation:
  (11) $$\theta_{\mathrm{DIP}}=0.67\theta_{\mathrm{PIP}}$$
  where *θ_DIP_* is the bending angle of a DIP joint and *θ_PIP_* is the bending angle of a proximal interphalangeal (PIP) joint.
- Angle range constraints
  This type of constraints refers to the limits of the ranges of finger motion as a result of hand anatomy. It is usually represented by the following equation:
  (12) $$\begin{array}{c} 0{}^{\circ}\leq \theta_{\mathrm{MCP}\_\mathrm{Flexion}}\leq 90^{\circ }\\ 0{}^{\circ}\leq \theta_{\mathrm{PIP}\_\mathrm{Flexion}}\leq (90{}^{\circ}∼100{}^{\circ})\\ 0{}^{\circ}\leq \theta_{\mathrm{Thumb}\_\mathrm{IP}\_\mathrm{Flexion}}\leq 90{}^{\circ}\\ \theta_{\mathrm{MCP}\_\mathrm{abduction}}=0{}^{\circ}\text{for the middle finger}\\ 0{}^{\circ}\leq \theta_{\mathrm{MCP}\_\mathrm{abduction}}\leq 30{}^{\circ}\text{for the other fingers} \end{array}$$
- One-axis constraints
  For DIP, PIP, and thumb interphalangeal (IP) joints, only one axis of movements is available. It means that the bending angle is the unique parameter to be measured.

### 3.1. The Hand Model

**Figure 4.**
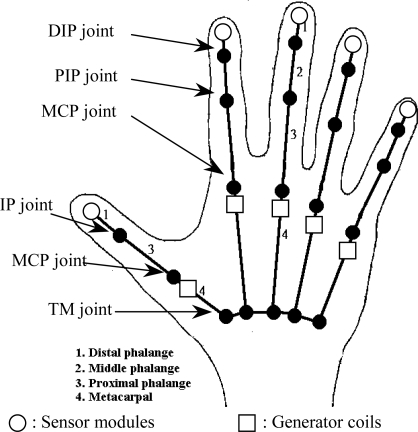
The hand model and the positions of the sensor modules and generator coils.

As mentioned in the above motion constraints of the hand, each of DIP, PIP, and thumb IP joints has only one DOF. It is also assumed that the MCP joint of the middle finger has only one DOF, but the MCP joints of the other fingers have two DOF; namely, MCP bending angles and abduction angles. Figure 4 describes the hand model and the positions of the sensor modules and generator coils of the data glove.

In this data glove, we measure the positions of five fingertips of the hand using five sensor modules and ten generator coils that are arranged into five groups. For the middle finger, one additional sensor coil is installed such that it is co-centered and perpendicular with the generator coils. This sensor coil is used for measuring three abduction angles called index-middle, ring-middle, and pinkie-ring abduction angles. By tracking fingertip positions, the object manipulation tasks in a virtual environment or teleoperation system can be carried out more precisely, because in most of grasping processes, fingertips are the foremost areas that reach the surface of an object. After the fingertip position has been determined, the shape of the associated finger can be modeled using the relationship between adjacent phalanges, and the corresponding bending angles of finger joints can be calculated, too. With this technique, our data glove has the ability of measuring 17 DOF of the hand. These bending angles are listed in Table 1.

### 3.2. Measuring the Fingertip Position

The data glove we develop contains sensor modules and generator coils that are placed on the fingertips and metacarpals of the hand, respectively. To make the measurement process easier, they must be installed in such a way, so that the orientation of the sensor module from the X*_L_*-axis is always equal to zero (*ϕ_c_* = 0). To achieve this, each generator coil must be made rotatable such that its rotational direction is consistent with the rotation of the abduction angle for the corresponding finger.

**Figure 5.**
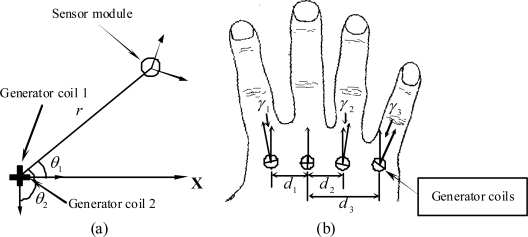
The positions and orientations of: (a) the sensor module and generator coils of one finger; (b) the generator coils of four fingers.

On the assumption that the metacarpal of a finger is laid on the **X**-axis, the position of a fingertip can be expressed by the distance *r* measured from the sensor module to the generator coils and the angle *θ_1_* measured from the **X**-axis. Two generator coils that are identical, perpendicular, and co-centered (the generator module) are used for measuring the above two parameters. They are placed on the metacarpal such that the normal vector of the first coil is parallel to the **X**-axis, as shown in Figure 5a.

Since the two generator coils are perpendicular, the orientation of the second generator coil can be written as:

(13) $$\theta_{2}=\theta_{1}+90{}^{\circ}$$

By substituting (13) into (10), the respective power *Emf* signals produced by the sensor module with respect to the first and the second generator coils can be expressed as:

(14) $$\begin{array}{c} {\mathrm{EmfM}}_{1}=\frac{2}{r^{6}}{\left(k\frac{\mathrm{dI}}{\mathrm{dt}}\right)}^{2}\left(3\;\text{cos}\;{2\theta }_{1}+5\right)\\ {\mathrm{EmfM}}_{2}=\frac{2}{r^{6}}{\left(k\frac{\mathrm{dI}}{\mathrm{dt}}\right)}^{2}\left(-3\;\text{cos}\;{2\theta }_{1}+5\right) \end{array}$$

Finally, the parameters *θ_1_* and *r* can be solved from (14), and are summarized in the following:

(15) $$\begin{array}{c} \theta_{1}=\frac{1}{2}\;{\text{cos}}^{-1}\;\left(\frac{5({\mathrm{EmfM}}_{1}-{\mathrm{EmfM}}_{2})}{3({\mathrm{EmfM}}_{1}+{\mathrm{EmfM}}_{2})}\right)\\ r=\sqrt[{6}]{\frac{20{\left(k\frac{\mathrm{dI}}{\mathrm{dt}}\right)}^{2}}{{\mathrm{EmfM}}_{1}+{\mathrm{EmfM}}_{2}}} \end{array}$$

### 3.3. Measuring the Abduction Angle

To measure the abduction angles of three fingers, one additional sensor coil is installed on the metacarpal of the middle finger. This sensor coil is also co-centered and perpendicular with the generator coils, which is shown in Figure 5(b). There are three abduction angles to be measured, including index-middle, ring-middle, and pinkie-ring abduction angles. The parameters *d_1_*, *d_2_* and *d_3_* used for calculating these three abduction angles are measured using the first generator coil of the middle finger during the calibration process. When measuring the associated abduction angle, only the first generator coil of the corresponding finger is activated; for example, to measure the index-middle abduction angle, only the first generator coil of the index finger is adopted. By applying (6), the *Emf* signal produced by the sensor coil of the middle finger can be expressed as:

(16) $$\begin{array}{c} {\mathrm{Emf}}_{n}=-\frac{k}{d_{n}^{3}}\;\frac{\mathrm{dI}}{\mathrm{dt}}\;(-\text{sin}\;\gamma_{n}\;(3\;\text{cos}\;2(\gamma_{n}+90{}^{\circ})+1)+3\;\text{cos}\;\gamma_{n}\;\text{sin}\;2(\gamma_{n}+90{}^{\circ}))=\frac{4k}{d_{n}^{3}}\;\frac{\mathrm{dI}}{\mathrm{dt}}\;\text{sin}\;\gamma_{n}\\ \text{for}\;\;n=1,\;2,\;3 \end{array}$$

where *Emf_1_*, *Emf_2_* and *Emf_3_* correspond to the outputs of the sensor coils with respect to the first generator coil of the index, ring, and pinkie fingers, respectively. The angle *γ_n_* can be solved from (16), which yields:

(17) $$\begin{array}{cc} \gamma_{n}={\text{sin}}^{-1}\;\left(\frac{{\mathrm{Emf}}_{n}\;d_{n}^{3}}{2k\frac{\mathrm{dI}}{\mathrm{dt}}}\right) & \;\;\;\;\text{for}\;\;n=1,\;2,\;3 \end{array}$$

Consequently, the three abduction angles can be determined below:

Index-middle abduction angle =*γ_1_*,
ring-middle abduction angle =*γ_2_*,
and pinkie-ring abduction angle =*γ_3_* *– γ_2_*.

### 3.4. Constructing the Shape of a Finger

**Figure 6.**
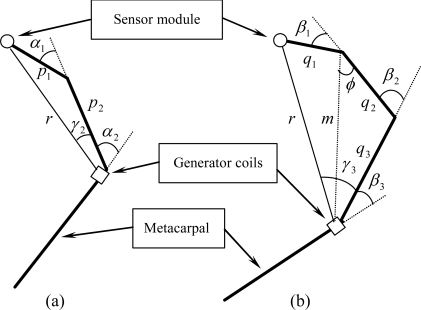
The relationship between the distance *r* and the bending angles of finger joints: (a) for the thumb; (b) for the other fingers.

The objective of constructing the shape of a finger is to estimate the bending angles of the DIP, PIP, and thumb IP joints. To accomplish this, only the distance *r* between the sensor module and the generator coils is involved, while the parameter *θ_1_* is used for estimating the bending angle of the MCP joint. Figure 6 illustrates the relationship between the distance *r* and the bending angles for the thumb and the other fingers.

The parameters *α_1_* and *α_2_* are the bending angles of the thumb IP and MCP joints, while *β_1_*, *β_2_*, and *β_3_* are the bending angles of the DIP, PIP, and MCP joints of the other fingers, respectively. The length of finger phalanges *p_1_*, *p_2_*, *q_1_*, *q_2_*, and *q_3_* are calculated during the calibration process. From Figure 5 and Figure 6, it is apparent that the bending angle of the MCP joint (*α_2_* and *β_3_*) can be calculated using the following equations:

(18) $$\alpha_{2}=\theta_{1}-\gamma_{2}\;\text{and}\;\alpha_{3}=\theta_{1}-\gamma_{3}$$

where:

(19) $$\begin{array}{c} \gamma_{2}={\text{cos}}^{-1}\;\left(\frac{r^{2}+p_{2}^{2}-p_{1}^{2}}{{2\mathrm{rp}}_{2}}\right)\\ \gamma_{3}={\text{cos}}^{-1}\;\left(\frac{r^{2}+q_{3}^{2}-q_{1}^{2}-q_{2}^{2}-2q_{1}q_{2}\;\text{cos}\;0.67\;\beta_{2}}{{2\mathrm{rq}}_{2}}\right) \end{array}$$

The bending angle of the thumb IP joint related to the distance *r* can be calculated directly using the following equation:

(20) $$\alpha_{1}={\text{cos}}^{-1}\;\left(\frac{r^{2}-p_{1}^{2}-p_{2}^{2}}{2p_{1}\;p_{2}}\right)$$

According to the motion constraints, the bending angles of the DIP joints of the index, middle, ring, and pinkie fingers are closely related to those of the PIP joints. With reference to Figure 6, this relationship can be further written as:

(21) $$\beta_{1}=0.67\;\beta_{2}$$

The distance *r* between the sensor module and the generator coils can be formulated as follows:

(22) $$\begin{array}{c} r^{2}=m^{2}+q_{1}^{2}+{2\mathrm{mq}}_{1}\;\text{cos}(0.67\;\beta_{2}+\phi )\\ =m^{2}+q_{1}^{2}+{2q}_{1}\;(q_{2}+q_{3}\;\text{cos}\;\beta_{2})\text{cos}\;0.67\;\beta_{2}-2q_{1}q_{3}\;\text{sin}\;0.67\;\beta_{2}\;\text{sin}\;\beta_{2}\\ =q_{1}^{2}+q_{2}^{2}+q_{3}^{2}+2q_{1}q_{2}\;\text{cos}\;0.67\;\beta_{2}+2q_{2}q_{3}\;\text{cos}\;\beta_{2}+2q_{1}q_{3}\;\text{cos}\;1.67\;\beta_{2} \end{array}$$

Therefore, the angle *β_2_* can be solved from (22), and thus the bending angles of DIP, PIP, and MCP joints can be calculated.

## 4. Data Glove Calibration

To make the data glove easy to use, the calibration process should be made as simple and efficient as possible. This means that there is no additional device needed; as a consequence, users merely wear the data glove during the calibration process.

**Figure 7.**
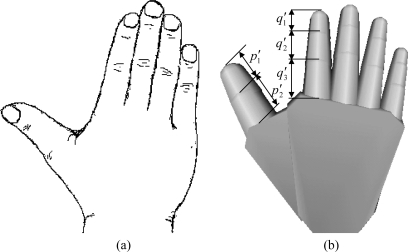
Illustration of a calibration gesture: (a) the pose of a real hand; (b) the corresponding virtual hand.

For our data glove, the calibration process consists of only one calibration gesture, as shown in Figure 7(a). The *Emf* signals produced by each sensor module and sensor coil are measured and stored into memory for further processing. When performing the calibration, the bending angle for each finger joint is equal to zero; accordingly, the parameters *θ_1_* and *r* will be equal to:

(23) $$\begin{array}{c} \theta_{1}=0{}^{\circ}\\ r=p_{1}+p_{2}\;\text{for the thumb and}\;r=q_{1}+q_{2}+q_{3}\;\text{for the other fingers} \end{array}$$

Then the parameters *p_1_*, *p_2_*, *q_1_*, *q_2_*, and *q_3_* can be calculated based on the proportional length of the finger phalanges from the virtual hand as Figure 7(b) shows. The equations of those parameters can be summarized as follows:

(24) $$\begin{array}{c} p_{1}=\frac{{\mathrm{rp}}_{1}^{\prime }}{p_{1}^{\prime }+p_{2}^{\prime }}\;\;\text{and}\;\;p_{2}=\frac{{\mathrm{rp}}_{2}^{\prime }}{p_{1}^{\prime }+p_{2}^{\prime }},\\ q_{1}=\frac{{\mathrm{rq}}_{1}^{\prime }}{q_{1}^{\prime }+q_{2}^{\prime }+q_{3}^{\prime }},\;\;q_{2}=\frac{rq_{2}^{\prime }}{q_{1}^{\prime }+q_{2}^{\prime }+q_{3}^{\prime }}\;\text{and}\;q_{3}=\frac{{\mathrm{rq}}_{3}^{\prime }}{q_{1}^{\prime }+q_{2}^{\prime }+q_{3}^{\prime }} \end{array}$$

In this calibration process, the first generator coil of the middle finger is also used as the sensor coil for measuring the parameters of the abduction angles. When a user is making the calibration gesture, the normal vectors of the sensor and generator coils will parallel such that the orientation parameters can be written as:

(25) $$\theta =90{}^{\circ}\;\text{and}\;\theta_{c}=0{}^{\circ}$$

The parameters of the abduction angle, that is, the distances between the sensor and generator coils, can be solved by substituting (25) into (6), which are stated in the following:

(26) $$\begin{array}{cc} d_{n}=\sqrt[{3}]{\frac{2k}{{\mathrm{Emf}}_{n}}\frac{\mathrm{dI}}{\mathrm{dt}}} & \text{for n}=1,2,3 \end{array}$$

## 5. Experimental Glove Sensor Results

To verify the validity of the derived *Emf* equations, experiments of bending angle, linear movement, and rotational angle measurements were performed. In those experiments, a signal generator circuit that produced a sinusoidal waveform was inputted to the generator coils, and the *Emf* signals generated by the sensor coil and sensor module were measured using an oscilloscope.

### 5.1. Bending Angle Experiment

**Figure 8.**
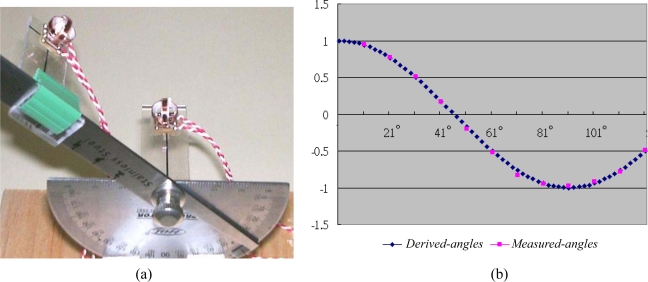
Illustration of a bending angle experiment: (a) the bending angle measurement device; (b) the derived angles *versus* the measured angles in the bending angle experiment.

In this experiment, the generator and sensor coils are placed on the bending angle measurement device as Figure 8 (a) shows. The generator coils are located on the rotational axis of the device, while the sensor module is placed on the moveable ruler, so that the distance between the sensor module and generator coils is adjustable. The power *Emf* signals produced by the sensor module with the rotational degrees ranging from 10° to 120° for *r* = 6 cm are recorded and listed in Table 2, where the constant *k* is equal to 1.2523 × 10^−5^ Hcm^3^. These measured values are used for calculating the cosine of the bending angles to compare with their derived values from (15) as follows:

(27) $$\begin{array}{c} \mathrm{Measured-value}=\frac{5({\mathrm{EmfM}}_{1}-{\mathrm{EmfM}}_{2})}{3({\mathrm{EmfM}}_{1}+{\mathrm{EmfM}}_{2})}\\ \mathrm{Derived-value}=\text{cos}{2\theta }_{1} \end{array}$$

Figure 8(b) plots the derived values *versus* the measured values of the bending angles. It is obvious that the measured values almost coincide with the theoretic values.

### 5.2. Linear Movement Experiment

In this experiment, the generator coils and sensor modules are also placed on the bending angle measurement device, as shown in Figure 8(a). The angle *θ*_1_ is set to 60°, while the distance *r* is varied from 5 to 10 cm.

**Figure 9.**
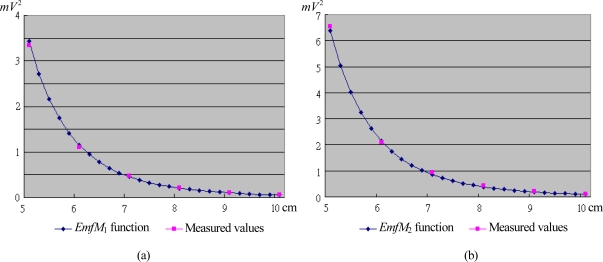
The power *Emf* function *versus* the measured values for: (a) *EmfM*_1_; (b) *EmfM*_2_ in the linear movement experiment.

The power *Emf* signals produced by the sensor module with the distance ranging from 5 to 10 cm from the generator coils are recorded and listed in Table 3, where the constant *k* is also equal to 1.2523 × 10^−5^ Hcm^3^. Figure 9 plots the power *Emf* function of (14) *versus* the measured values. We can see that the measured values perfectly match the power *Emf* function.

### 5.3. Rotational Angle Experiment

**Figure 10.**
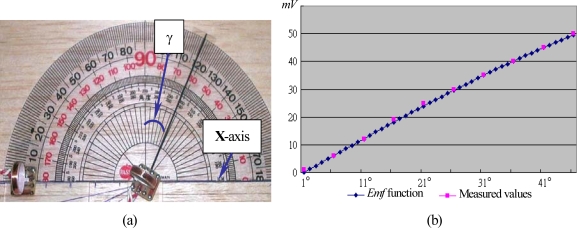
Illustration of a bending angle experiment: (a) the rotational angle measurement device; (b) the *Emf* function *versus* the measured values.

In this experiment, both sensor and generator coils are placed on the rotational angle measurement device, as shown in Figure 10 (a). The generator coil is rotatable, while the sensor coil is fixed on the X-axis with the distance equal to 5 cm from the generator coil. The constant *k* is equal to 0.995957 × 10^−5^ Hcm^3^. The *Emf* signals produced by the sensor coil with the rotational angle γ from 0° to 45° are recorded and listed in Table 4. Figure 10 (b) plots the *Emf* function of (16) *versus* the measured values. Apparently, the measured values flawlessly match the *Emf* function.

## 6. Realization of the Data Glove System

The data glove we have developed applies generator coils to generate electromagnetic signals. The sensor coil and sensor modules arranged on the metacarpal of the middle finger and fingertips, respectively, sense the electromagnetic signals and then measure the generated *Emf* signals. Based on the measured *Emf* signals, the abduction angles and fingertip positions of the hand are calculated using the derived equations as formulated in Section 3. Since ten generator coils are installed on the data glove, we should pay attention to the prevention of interference among them. There are three commonly used techniques for solving this problem:

- Frequency division method
  Each generator coil is driven by a sinusoidal waveform of different frequencies and the outputs of the sensor coil and sensor module are bandpass-filtered to get an appropriate *Emf* signal.
- Time division method
  In this technique, only one sinusoidal signal is used to drive the generator coils. To prevent interference, each generator coil is given an activation time slice and scanned on a round-robin basis. Within each time slice, the outputs of the associated sensor coil and sensor module are detected and the resulting analog streams are fed to an A/D converter.
- Mix of frequency and time division method
  With this method, two frequency bands of sinusoidal signals can be applied to activating the generator coils. Such generator coils are organized into five groups corresponding to each finger of the hand. Then, these five groups of generator coils are controlled by the time division method.

**Figure 11.**
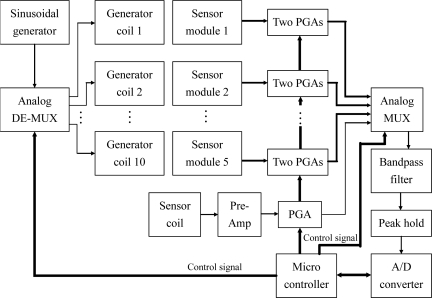
The block diagram of the data glove control system.

Both the time division and mix of frequency and time division methods are feasible for developing the prototype of our data glove, because the sinusoidal signals that drive the generator module must have similar characteristics. In this prototype, we adopt the time division method to control the generator and sensor coils. The advantage of this method is the feasibility of controlling them entirely via digital circuits. The block diagram of the data glove control system is shown in Figure 11.

**Figure 12.**
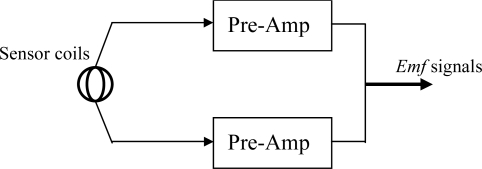
The block diagram of the sensor module.

The sinusoidal generator circuit will continuously produce a sinusoidal signal that is fetched into an analog demultiplexer. The micro controller unit will generate timing and control signals for selecting an appropriate generator coil and enabling the output of the associated sensor module or sensor coil followed by a pre-amplifier to be passed to a bandpass filter by sending a control signal to an analog multiplexer. Before entering the analog multiplexer, the outputs of the pre-amplifier and sensor modules are fetched into the respective programmable-gain amplifiers for further amplification. Because we are only interested to the intensity of the *Emf* signal produced by the sensor coil, a peak hold circuit is connected with the output of the bandpass filter for tracking the maximum value of the sinusoidal signal generated by the sensor coil. The output of the peak hold circuit is a DC level signal originated from the maximum *Emf* signal of the sensor output. This DC level signal is then sampled by an A/D converter and stored into temporary memory in the micro controller unit. All the sensor outputs are sent to a host computer through a parallel communication port. The sensor module shown in Figure 12 consists of two sensor coils that are perpendicular and co-centered. The two pre-amplifiers are used for signal enhancement.

**Figure 13.**
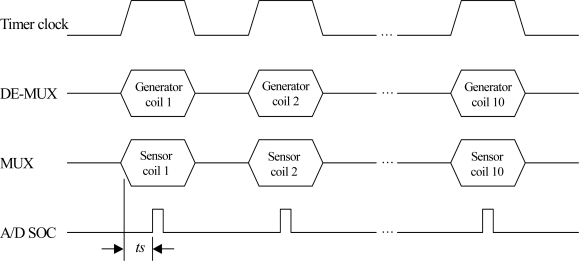
The timing diagram of the data glove control signals.

In the development of this data glove, we use the 16-bit microprocessor SPCE061 with a built-in 10-bit A/D converter provided by Sunplus Technology. Figure 13 depicts the timing diagram of the control signals.

The A/D SOC in Figure 13 is the start of the conversion signal for the A/D converter, where *ts* is the setup time required by the generator and sensor coils to produce a stable *Emf* signal. Table 5 lists the mapping pairs of the generator and sensor coils in the scanning process.

**Figure 14.**
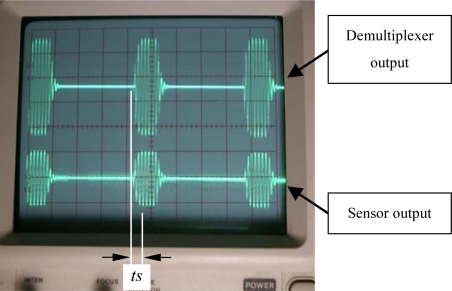
Sinusoidal output waveforms of the demultiplexer and the sensor coil.

Figure 14 illustrates the sinusoidal output waveform from the analog demultiplexer for a generator coil as well as the sinusoidal output waveform of the associated sensor coil with the time division method. To get a clear inspection on the sinusoidal waveform, in this demonstration, only two generator coils are scanned on a round-robin basis.

## 7. Conclusions

In this paper, the development of a data glove with fingertip tracking technology based on magnetic induction has been presented. This data glove has the ability of measuring 17 DOF by reconstructing the shape of the fingers after the positions and orientations of the fingertips have been estimated. By tracking the fingertips of the hand, the object manipulation tasks in a virtual environment or teleoperation system can be controlled more precisely, since in most of grasping processes, fingertips are the foremost areas that reach the surface of an object. To track the fingertips, we use five sensor modules and one sensor coil that are attached on the fingertips and metacarpal of the middle finger of a hand. The sensors and generators constructed with small magnetic induction coils are called the sensor coils and generator coils of the data glove, respectively. The sensor module consists of two sensor coils that are perpendicular and co-centered. All of these sensor and generator coils are placed on the fingertips and metacarpal positions of the hand, so that there is no direct contact point between the sensors and the finger joints. It means that the data glove sensors do not change their shapes when the fingers are bending. Thus, not only the quality of measurement is greatly improved, but also the lifetime of the sensors is greatly prolonged.

**Figure 15.**
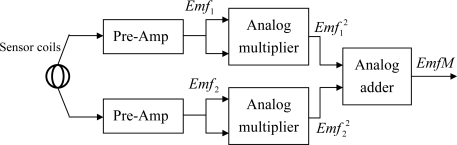
Hardware implementation of the sensor module.

To facilitate the action of the data glove, the sensor module can be constructed entirely by hardware using the currently available semiconductor technology, so that the power *Emf* signal is calculated directly using hardware as shown in Figure 15. Thus, more accurate results can be gained due to a higher SNR of the signal in the transmission wire.

The motion constraints applicable to simplifying the development of the data glove are also investigated. They are classified into three categories: intrafinger constraints, angle range constraints, and one-axis constraints. With the intrafinger constraints, the shape of a finger can be constructed based on the measured fingertip position, and hence, the DIP and PIP bending angles can be calculated. The angle range and one-axis constraints are devoted to simplify the *Emf* equations, so that the fingertip position and orientation can be easily calculated. For the convenience of using the data glove, we also propose a simple and efficient calibration process that comprises only one calibration gesture.

The theoretical formulation of the fingertip position and orientation, and the abduction angles of the finger joints as well as the calibration equations are derived directly from the positions and orientations of the generator and sensor coils on the data glove. Beside this, the circuit block diagram and the construction of the data glove system are described and realized. To prevent the interference among the generator coils, three methods of scanning the generator and sensor coils, including the frequency division, time division, and mix of frequency and time division techniques, are also discussed. After the evaluation, we adopted the time division method to control the generator and sensor coils in developing the prototype of our data glove. The advantage of this method is the feasibility of controlling them entirely via digital circuits. Another superiority of this data glove compared to use the Pohemus motion tracking system [21] is that the distances between the generator coils and sensor modules are close enough such that only small power of electromagnetic radiation is needed, and the interference of a metallic object with the distance greater than 20 cm from the data glove can be ignored. But the supporting of the Pohemus motion tracking system for detecting the positions of hands in a 3D environment becomes significantly important when the complete movements of hands are required. So far, the experimental results of the sensors performing linear movement and bending angle measurements are very satisfactory. It reveals that our data glove can act as an practical man-machine interface.

## Figures and Tables

**Table 1.** The list of seventeen bending angles.

**Bending Angles**			
Index PIP	Index MCP	Index DIP	Index-middle abduction
Middle PIP	Middle MCP	Middle DIP	Ring-middle abduction
Ring PIP	Ring MCP	Ring DIP	Pinkie-ring abduction
Pinkie PIP	Pinkie MCP	Pinkie DIP	
Thumb IP	Thumb MCP		

**Table 2.** The power *Emf* signal produced by a sensor module in the bending angle experiment.

*θ*_1_	*EmfM*_1_ (*mV*^2^)	*EmfM_2_* (*mV*^2^)
10°	2.690	0.746
20°	2.466	0.900
30°	2.152	1.143
40°	1.773	1.446
50°	1.354	1.731
60°	1.100	2.100
70°	0.833	2.500
80°	0.745	2.700
90°	0.654	2.500
100°	0.720	2.500
110°	0.867	2.400
120°	1.200	2.200

**Table 3.** The power *Emf* signal produced by a sensor module in the linear movement experiment.

Distance *r* (cm)	*EmfM*_1_ (*mV*^2^)	*EmfM*_2_ (*mV*^2^)
5	3.334	6.548
6	1.100	2.100
7	0.468	0.942
8	0.216	0.420
9	0.111	0.205
10	0.063	0.114

**Table 4.** The *Emf* signal produced by a sensor coil in the rotational angle experiment.

*γ*	*Emf* (*mV*)	*γ*	*Emf* (*mV*)
0°	1	25°	30
5°	6	30°	35
10°	12	35°	40
15°	19	40°	45
20°	25	45°	50

**Table 5.** The mapping pairs of the generator and sensor coils.

Generator coil	Sensor coil	Generator coil	Sensor coil
1	1	6	6
2	2	7	7, 11
3	3, 11	8	8
4	4	9	9, 11
5	5	10	10

## References

[1] Tung C.P., Kak A.C. (1995). Automatic learning of assembly tasks using a data glove system.

[2] Hashimoto H., Ogawa H., Umeda T., Obama M., Tatsuno K. (1995). An unilateral master-slave hand system with a force-controlled slave hand.

[3] Sing B.K., Ikeuchi K. (1995). A robot system that observes and replicates grasping tasks.

[4] Greenleaf W.J. (1995). Rehabilitation, ergonomics, and disability solutions using virtual reality technology.

[5] Satava R.M. (1995). Medicine 2001: the king is dead.

[6] Kawamura S., Ida M., Wada T., Wu J.L. (1995). Development of a virtual sports machine using a wire drive system—a trial of virtual tennis.

[7] Gromala D., Sharir Y. (1994). Dancing with the virtual dervish: virtual bodies’.

[8] Jaques M.W.S., Strickland P., Oliver T.J. (1994). Design by manufacturing simulation: concurrent engineering meets virtual reality.

[9] Jaques M.W.S., Harrison D.J. (1994). Using gestures to interface with a ‘virtual manufacturing’ package.

[10] Rosenberg R., Slater M. (1999). The chording glove: a glove-based text input device. IEEE Trans. System Man Cybern. Part C: Appli. Rev.

[11] Cho M.C., Park K.H., Hong S.H., Jeon J.W., Lee S.L., Choi H., Choi H.G. (2002). A pair of braille-based chord gloves.

[12] Babic R.V. (2002). Sensorglove—a new solution for kinematic virtual keyboard concept.

[13] Sturman D.J., Zeltzer D. (1994). A survey of glove-based input. IEEE Comput. Graphics Appli.

[14] Toney A. (1998). A novel method for joint motion sensing on a wearable computer.

[15] Perng J.K., Fisher B., Hollar S., Pister K.S.J. (1999). Acceleration sensing glove (ASG).

[16] Dogramadzi S., Allen C.R., Bell G.D., Rowland R. (1999). An electromagnetic imaging system for remote sign language communication.

[17] Tarchanidis K.N., Lygouras J.N. (2001). Data glove with a force sensor.

[18] Fahn C.S., Sun H. (2000). Development of a sensory data glove using neural-network-based calibration. Jour. Comput.

[19] Lin J., Wu Y., Huang T.S. (2000). Modeling the constraints of human hand motion.

[20] Sun H., Fahn C.S. (2007). Development of a virtual keyboard based on button tracking using magnetic induction. Jour. Chinese Inst. Eng.

[21] Polhemus http://www.polhemus.com/?page=Motion_Case_Studies_AMM.

